# Human Skin, Oral, and Gut Microbiomes Predict Chronological Age

**DOI:** 10.1128/mSystems.00630-19

**Published:** 2020-02-11

**Authors:** Shi Huang, Niina Haiminen, Anna-Paola Carrieri, Rebecca Hu, Lingjing Jiang, Laxmi Parida, Baylee Russell, Celeste Allaband, Amir Zarrinpar, Yoshiki Vázquez-Baeza, Pedro Belda-Ferre, Hongwei Zhou, Ho-Cheol Kim, Austin D. Swafford, Rob Knight, Zhenjiang Zech Xu

**Affiliations:** a Center for Microbiome Innovation, Jacobs School of Engineering, University of California, San Diego, La Jolla, California, USA; b UCSD Health Department of Pediatrics, University of California, San Diego, La Jolla, California, USA; c IBM T. J. Watson Research Center, Yorktown Heights, New York, USA; d IBM Research UK, The Hartree Centre, Warrington, United Kingdom; e Division of Biostatistics, University of California, San Diego, La Jolla, California, USA; f UCSD Division of Gastroenterology, University of California, San Diego, La Jolla, California, USA; g VA San Diego Health Care, La Jolla, California, USA; h Division of Laboratory Medicine, Zhujiang Hospital, Southern Medical University, Guangzhou, China; i Scalable Knowledge Intelligence, IBM Research-Almaden, San Jose, California, USA; j Department of Computer Science and Engineering, University of California, San Diego, La Jolla, California, USA; k Department of Bioengineering, University of California, San Diego, La Jolla, California, USA; l State Key Laboratory of Food Science and Technology, Nanchang University, Nanchang, China; m Biomedical Sciences Graduate Program, University of California, San Diego, La Jolla, California, USA; University of Pennsylvania

**Keywords:** age prediction, gut microbiota, oral microbiota, random forests, skin microbiota

## Abstract

Human gut microbiomes are known to change with age, yet the relative value of human microbiomes across the body as predictors of age, and prediction robustness across populations is unknown. In this study, we tested the ability of the oral, gut, and skin (hand and forehead) microbiomes to predict age in adults using random forest regression on data combined from multiple publicly available studies, evaluating the models in each cohort individually. Intriguingly, the skin microbiome provides the best prediction of age (mean ± standard deviation, 3.8 ± 0.45 years, versus 4.5 ± 0.14 years for the oral microbiome and 11.5 ± 0.12 years for the gut microbiome). This also agrees with forensic studies showing that the skin microbiome predicts postmortem interval better than microbiomes from other body sites. Age prediction models constructed from the hand microbiome generalized to the forehead and vice versa, across cohorts, and results from the gut microbiome generalized across multiple cohorts (United States, United Kingdom, and China). Interestingly, taxa enriched in young individuals (18 to 30 years) tend to be more abundant and more prevalent than taxa enriched in elderly individuals (>60 yrs), suggesting a model in which physiological aging occurs concomitantly with the loss of key taxa over a lifetime, enabling potential microbiome-targeted therapeutic strategies to prevent aging.

**IMPORTANCE** Considerable evidence suggests that the gut microbiome changes with age or even accelerates aging in adults. Whether the age-related changes in the gut microbiome are more or less prominent than those for other body sites and whether predictions can be made about a person’s age from a microbiome sample remain unknown. We therefore combined several large studies from different countries to determine which body site’s microbiome could most accurately predict age. We found that the skin was the best, on average yielding predictions within 4 years of chronological age. This study sets the stage for future research on the role of the microbiome in accelerating or decelerating the aging process and in the susceptibility for age-related diseases.

## Abstract (precis)

Considerable evidence suggests that the gut microbiome changes with age or even accelerates aging in adults. Whether the age-related changes in the gut microbiome are more or less prominent than those for other body sites and whether predictions can be made about a person’s age from a microbiome sample remain unknown. We therefore combined several large studies from different countries to determine which body site’s microbiome could most accurately predict age. We found that the skin was the best, on average yielding predictions within 4 years of chronological age. This study sets the stage for future research on the role of the microbiome in accelerating or decelerating the aging process and in the susceptibility for age-related diseases.

## OBSERVATION

Microbiomes across the body are known to change rapidly in the first 3 years of life and then relatively little in adults (1). Recent work suggests that the gut microbiome can be used to classify adults into age groups, with sex-specific differences in patterns of diversity by age (2). Age has been implicated as a dominant factor in the adult microbiome in numerous cohort studies (3–5). The microbiome also continues to change after death and has been used to predict postmortem interval to within a few days in both mice (6) and humans (7): contrary to expectation, the skin microbiome predicted postmortem interval much better than did the gut or the surrounding soil microbiome.

We were inspired by these results to expand our past work on age prediction in the gut microbiome (2) to other body sites, including the mouth and the skin. We used a total of 4,434 fecal samples (United States, *n* = 1,887; United Kingdom, *n* = 685; China, *n* = 1,609; others), 2,550 saliva samples (United States, *n* = 1,666; United Kingdom, *n* = 48; Tanzania, *n* = 254; others as well) (3, 8–11), and 1,975 skin samples (United States, *n* = 1,723; United Kingdom, *n* = 27; others) (3, 8, 9, 12). In total, this represents the most comprehensive investigation of microbiome and age, with 8,959 samples from 10 studies (3, 8–14).

We acquired 100-bp amplicon sequence variants (ASVs) processed with Deblur (15) from the 16S-V4 rRNA gene amplicon data in Qiita (16) using the redbiom search engine (17). This study includes only subjects with self-reported ages from 18 to 90 years (see Fig. S1 in the supplemental material), body mass indices (BMI) of 18.5 to 30 kg/m^2^, no reported inflammatory bowel disease or diabetes, and no antibiotic consumption 1 month before sampling. We also excluded pregnant, hospitalized, disabled, or critically ill individuals (Table S1). For gut microbiota, the majority of acquired samples were derived from two projects: (i) the American Gut Project (AGP) (3) and (ii) the Guangdong Gut Microbiome Project (GGMP) (13). For oral and skin microbiota, we obtained all samples from Qiita matching the inclusion and exclusion criteria above, representing the most comprehensive meta-analysis for age prediction using human microbiota. We further analyzed the ASV data with the QIIME 2 pipeline (18).

10.1128/mSystems.00630-19.1 FIG S1 The age distribution of all human individuals in the gut, oral, and skin microbiota data sets and its potential effect on age prediction. (A) The age distribution of all hosts. (B to E) The effect of skewed age distribution on the prediction model of the oral and skin microbiota. For comparison to the original data (B and D), we subsampled microbiota data from the young ages for age prediction using the random forest model (C and E), where we balanced the sample size of young (<40 years) and old (>40 years) individuals. The scatterplots related to the subsampled data show that either human oral or skin microbiota age can be highly predictive in young adults but have less strong association with age in older adults. Download FIG S1, PDF file, 0.3 MB. Copyright © 2020 Huang et al. 2020 Huang et al. This content is distributed under the terms of the Creative Commons Attribution 4.0 International license.

10.1128/mSystems.00630-19.5 TABLE S1 Statistical summary of data meeting inclusion criteria for our analysis. Download Table S1, XLSX file, 0.01 MB. Copyright © 2020 Huang et al. 2020 Huang et al. This content is distributed under the terms of the Creative Commons Attribution 4.0 International license.

We used random forests (RF) (19) to regress relative abundances of ASVs in the healthy human microbiota from different body sites (gut, oral, and skin microbiota) against the subjects’ chronological ages with the R package ranger (20) using fine-tuned hyperparameters. To test if confounders (such as sex) affected the modeling, we first trained the age model within a sub-data set stratified by a confounder and then applied it on all the other sub-data sets. For both model training and testing, we evaluated regression performance using mean absolute error (MAE). We fit a smoothing spline function between microbiota age and chronological age to calculate relative microbiota age. Relative microbiota age per sample was calculated as the difference between the microbiota age of a focal adult and the microbiota age of the interpolated spline fit of healthy adults at the same chronological age. We used the Wilcoxon rank sum test (21) to compare relative microbiota ages between host groups in each data set. To determine the effects of country and body sites on microbiota age, we subdivided the data sets into these groups and repeated the analyses.

**FIG 1:**
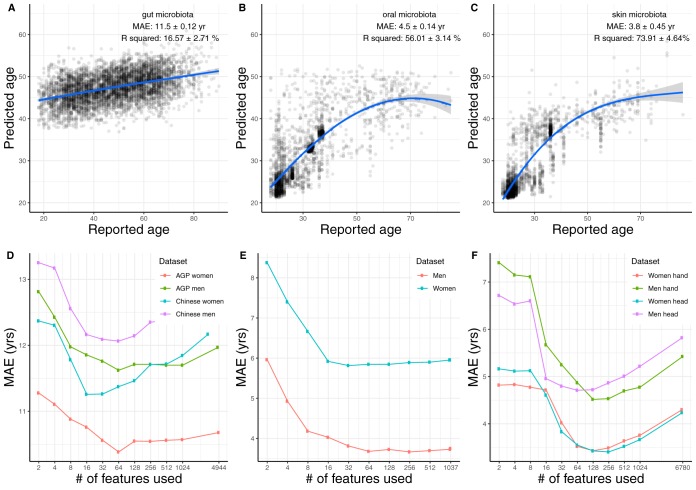
The distinct capability for age prediction from gut (A), oral (B), and skin (C) microbiomes. Spline fit to the data is also shown (blue curve). Although the skewed age distribution in the skin or oral microbiota data set may decrease the accuracy of age prediction for the older adults, it will not affect the conclusions about the relative abilities of different human microbiomes to predict age. Prediction performances at increasing numbers of microbial species were obtained by retraining the random forest classifier on the top-ranking features (ASVs), shown in terms of mean absolute error (MAE) from gut (D), oral (E), and skin (F) microbiota identified with previous random forest models trained in different cohorts. Data are from Qiita studies 11757, 10317, 550, 1841, 1774, 2010, 2024, 2202, 11052, and 10052.

The RF regression recaptured the known result that the gut microbiome is associated with chronological age (Fig. 1A) and that this relationship holds across cohorts, but the connections to age were even stronger in the oral (Fig. 1B) and skin (Fig. 1C) microbiomes. Remarkably, the skin microbiome could pinpoint a subject’s age to within 4 years, on average. Analysis of the specific microbial features contributing to these models demonstrated that relatively few ASVs (e.g., around 64) are needed for highly accurate models for each body site (Fig. 1D to F).

We next tested whether the models were sex specific. As shown in previous work (2), we found a sex-specific signal in the gut microbiome; however, we did not find a sex-specific signal in the mouth or the skin microbiome. Consequently, although we observed a small degradation in prediction accuracy for the cross-trained models from men to women or vice versa for the gut, we saw no such degradation for the other body sites, suggesting that populations do not need to be stratified by sex to build such predictive models.

**FIG 2:**
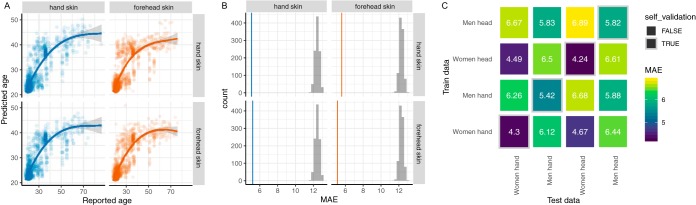
The skin microbiota age prediction model can be applied across forehead and hand microbiota. (A) The microbiota age of hand (orange) or forehead (blue) as calculated by a random forest model trained on the hand (upper scatterplots) or forehead (lower scatterplots) subsets; lines indicate spline fit. (B) The prediction accuracy of age regression models dependent on skin body sites and their cross-applications compared to random permutations. The vertical lines indicate the prediction accuracy (mean absolute error) of age models trained in forehead (orange) or hand (blue) sites and their testing on the other site, while the gray histograms show the MAE distribution in 1,000 permutations of age values in either training or testing data. (C) Cross-prediction matrix reporting prediction performances as MAE values obtained using a random forest model on ASV relative abundances. Matrix values refer to the MAE values obtained by training the regressor on the data set of the corresponding row and applying it to the data set of the corresponding column. The prediction accuracies between sexes are higher than those between body sites.

For the skin, we had enough data for the forehead and palm to test whether models trained on skin from one body site apply for the other. This is important because the forehead and hand are markedly different in physiology and microbiology (22). Figure 2A and B demonstrate that models of microbiome age for the forehead can be cross-trained on the palm, and the converse is also true (Fig. 2C). This means that future studies seeking to determine factors leading to microbiome aging can combine these skin sites, which is important given the tremendous microbiome and metabolic diversity observed across the human body (23).

An important consideration is which taxa contribute to the age prediction model. In the gut, the ASVs belonging to the genera *Bifidobacterium* and *Blautia* or the families *Lachnospiraceae*, *Ruminococcaceae*, and *Clostridiaceae* consistently had high feature importance scores, although values differed between populations and within populations (Fig. S2). A larger discrepancy in feature importance rankings was found between aging models built from different countries. For example, the top-ranking feature in Chinese cohorts is an ASV belonging to *Bifidobacterium*, but it was not detectable in the U.S. cohort. In the oral microbiota, we identified a set of top-ranking microbial markers decreasing in abundance with host aging in both females and males, such as ASVs belonging to *Lactobacillales*, *Gemellaceae*, *Bacteroides*, and *Fusobacterium* (Fig. S3). In the skin microbiome, we identified age-related markers in four subgroups: female forehead, male forehead, female palm, and male palm. As we age, changes in skin physiology (such as decreased sebum production and increased dryness) and host immune system can alter associated microbiota (24–26). Interestingly, we identified several genera and families that include anaerobic members (i.e., ASVs belonging to *Mycoplasma*, *Enterobacteriaceae*, and *Pasteurellaceae*) negatively correlated with age in all subgroups, reflecting these physiological changes due to aging (Fig. S4).

10.1128/mSystems.00630-19.2 FIG S2 Ranking relevance of each important feature (ASV) in the predictive models (AGP women, AGP men, Chinese women, and Chinese men) in the gut data set. The importance of each ASV for the cross-validation prediction performance in each data set was estimated using the internal random forest scores. Only features among the 64 top-ranking features in at least one data set are reported. Download FIG S2, EPS file, 0.04 MB. Copyright © 2020 Huang et al. 2020 Huang et al. This content is distributed under the terms of the Creative Commons Attribution 4.0 International license.

10.1128/mSystems.00630-19.3 FIG S3 Ranking relevance of each importance feature (ASV) in the predictive models (women and men) in the oral data set. The importance of each ASV for the cross-validation prediction performance in each data set was estimated using the internal random forest scores. Only features among the 64 top-ranking features in at least one data set are reported. Download FIG S3, EPS file, 0.04 MB. Copyright © 2020 Huang et al. 2020 Huang et al. This content is distributed under the terms of the Creative Commons Attribution 4.0 International license.

10.1128/mSystems.00630-19.4 FIG S4 Ranking relevance of each top important feature (ASV) in the predictive models (female head, male head, female hand, and male hand) in the skin data set. The importance of each ASV for the cross-validation prediction performance in each data set was estimated using the internal random forest scores. Only features among the 64 top-ranking features in at least one data set are reported. Download FIG S4, EPS file, 0.04 MB. Copyright © 2020 Huang et al. 2020 Huang et al. This content is distributed under the terms of the Creative Commons Attribution 4.0 International license.

These results are consistent with city-specific influences on models for predicting clinical states (13). However, the success of the cross-population generalization suggests that the types of tuned RF models we introduce here may result in robust and generalizable predictors. Interestingly, gut and oral bacteria that are enriched in young individuals are both more abundant and more prevalent than bacteria enriched in elderly individuals. We calculated the average relative abundance and ubiquity for each ASV in the shared response. We found that bacteria that are enriched in young individuals in at least two cohorts are both ubiquitous and abundant across people, whereas those enriched in old age are less abundant and not ubiquitous. Thus, the presence of these ASVs enriched in elderly individuals is a good indicator of microbial shifts associated with aging.

Taken together, our results demonstrate that accurate and generalizable indicators of age can be derived from microbiome studies using machine learning techniques and that prediction is most accurate from the skin microbiome. Building on these results, future work will include developing noninvasive microbiome-based tests to determine signs of accelerated or delayed aging in the elderly, or in individuals with chronic diseases, and designing and evaluating microbially based interventions to modify the aging process.

### Data availability.

The data and code for this study are available at https://github.com/shihuang047/age-prediction.
