# Supplementary figures and images for: Human Skin, Oral, and Gut Microbiomes Predict Chronological Age
Source: mSystems. 2020 Feb 11;5(1):e00630-19. doi: 10.1128/mSystems.00630-19 (PMC7018528; doi:10.1128/mSystems.00630-19)

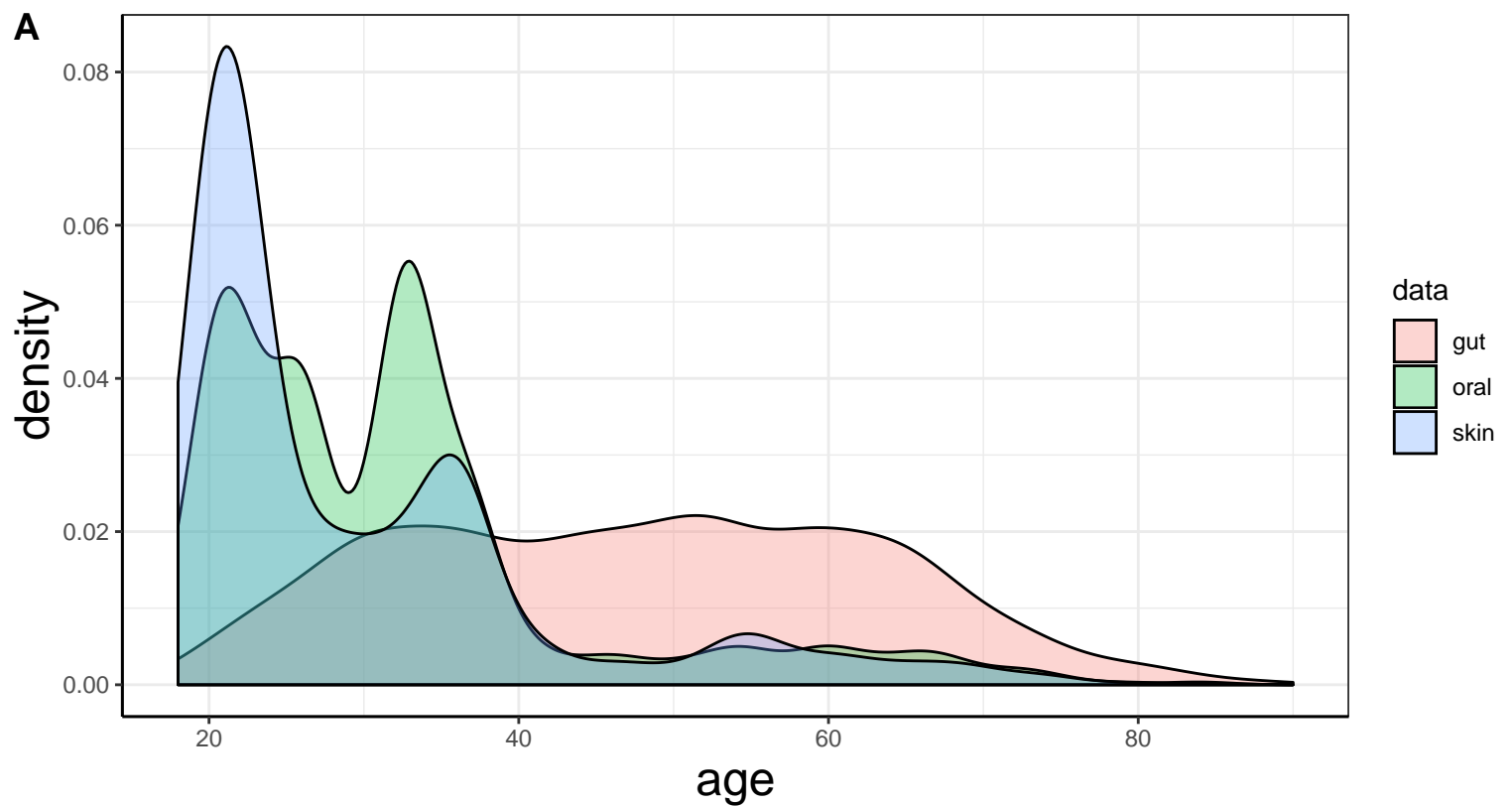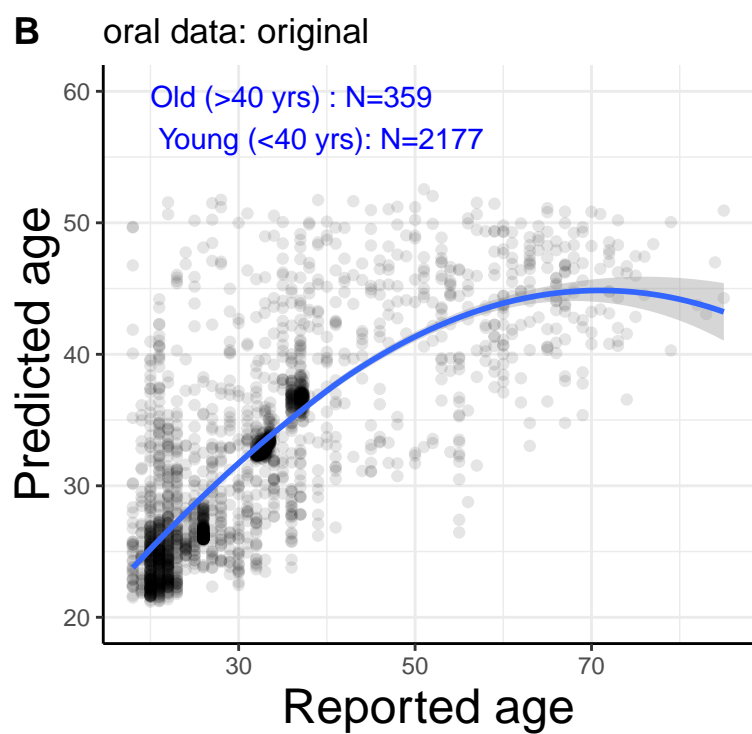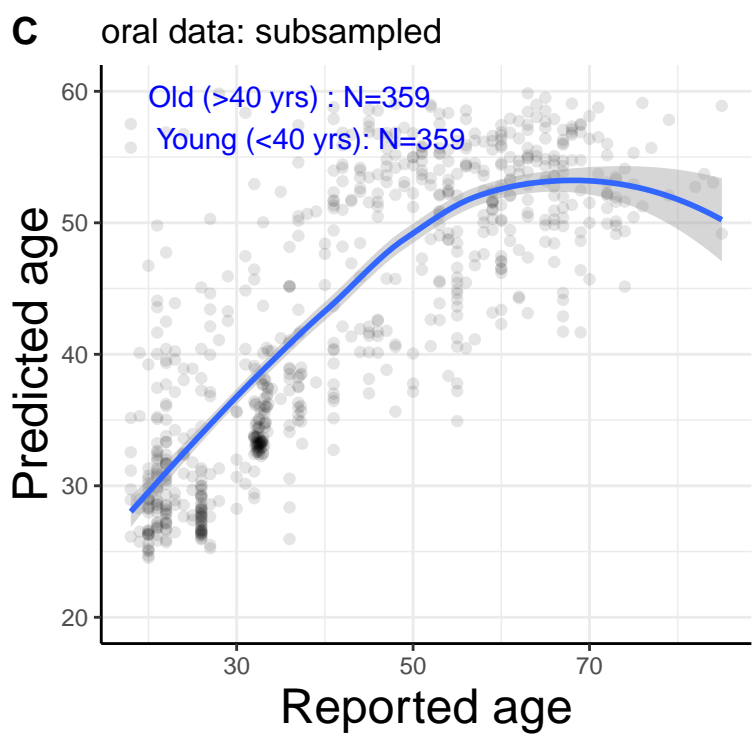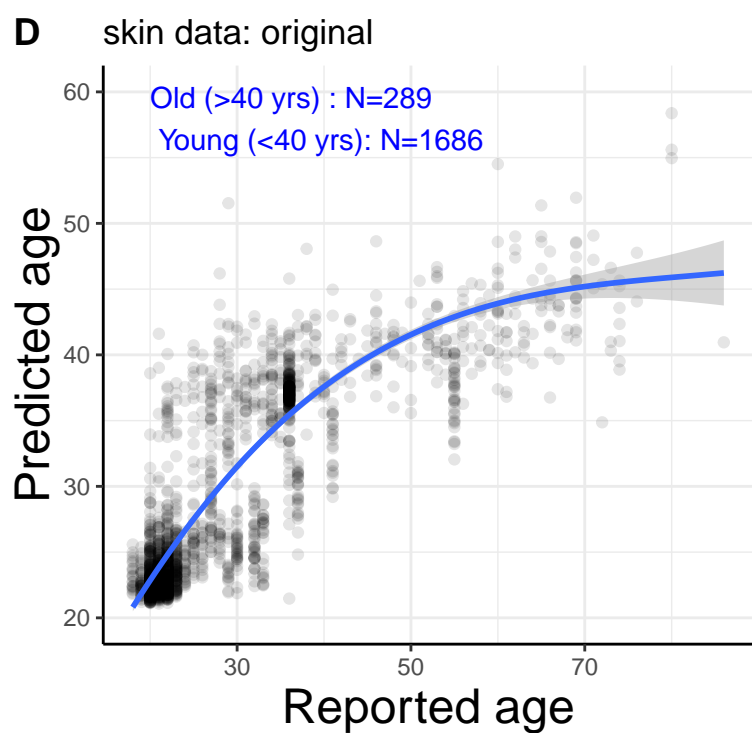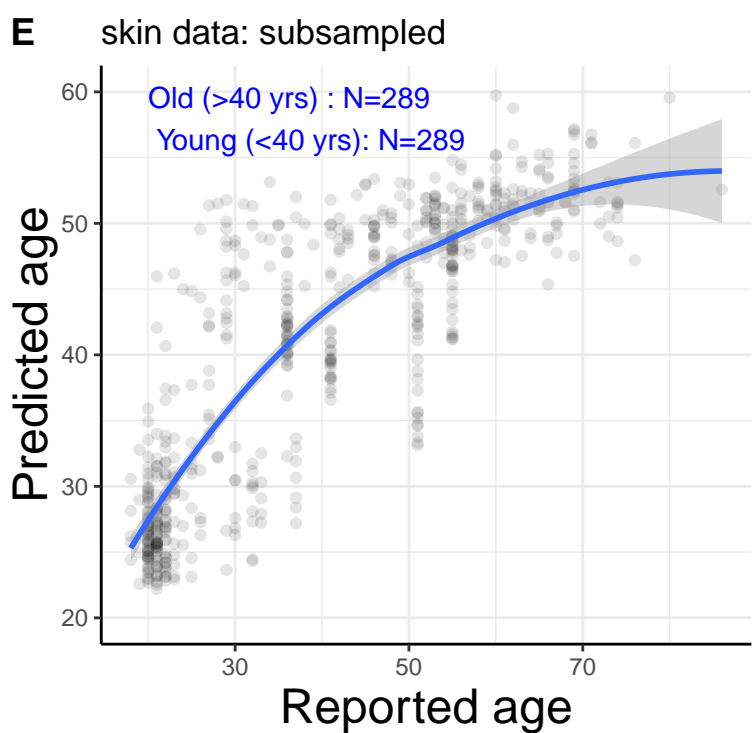

Supplement: FIG S1 [file mSystems.00630-19-sf001.pdf]
